# Fingerprinting shock-induced deformations via diffraction

**DOI:** 10.1038/s41598-021-88908-y

**Published:** 2021-05-10

**Authors:** Avanish Mishra, Cody Kunka, Marco J. Echeverria, Rémi Dingreville, Avinash M. Dongare

**Affiliations:** 1 Department of Materials Science and Engineering, University of Connecticut, Storrs, CT 06269 USA; 2 Institute of Materials Science, University of Connecticut, Storrs, CT 06269 USA; 3 Center for Integrated Nanotechnologies, Sandia National Laboratories, Albuquerque, NM 87123 USA

**Keywords:** Atomistic models, Mechanical properties

## Abstract

During the various stages of shock loading, many transient modes of deformation can activate and deactivate to affect the final state of a material. In order to fundamentally understand and optimize a shock response, researchers seek the ability to probe these modes in real-time and measure the microstructural evolutions with nanoscale resolution. Neither post-mortem analysis on recovered samples nor continuum-based methods during shock testing meet both requirements. High-speed diffraction offers a solution, but the interpretation of diffractograms suffers numerous debates and uncertainties. By atomistically simulating the shock, X-ray diffraction, and electron diffraction of three representative BCC and FCC metallic systems, we systematically isolated the characteristic fingerprints of salient deformation modes, such as dislocation slip (stacking faults), deformation twinning, and phase transformation as observed in experimental diffractograms. This study demonstrates how to use simulated diffractograms to connect the contributions from concurrent deformation modes to the evolutions of both 1D line profiles and 2D patterns for diffractograms from single crystals. Harnessing these fingerprints alongside information on local pressures and plasticity contributions facilitate the interpretation of shock experiments with cutting-edge resolution in both space and time.

## Introduction

Understanding and optimizing a shock response requires characterization not only of the final state but also of the transient microstructures that form during the propagation of the compression wave, the release wave, and their interactions leading to that final state. Over only a few tens of picoseconds, several deformation modes can activate and deactivate (even simultaneously) to significantly alter the material down to the nanoscale. Moreover, the relative contributions and timings of these deformation modes can differ dramatically depending on the material. In this manuscript, we examined three representative metallic systems in order to feature three of the most important deformation modes for shocked FCC and BCC microstructures: dislocation slip (stacking faults), deformation twining, and phase transformation. When shocking Ta along [110], twins form during the compressive wave and are annihilated by the release wave^1–9^. In contrast, when shocking Fe along [110], the compressive wave induces an α (BCC) → ϵ (HCP) phase transformation, and then the release wave reverses this transformation and induces twins^10–13^. Finally, when shocking Cu along [111], stacking faults rather than twins or phase transformations dominate the deformation response^9,14–17^.

Because these deformation modes operate at the picosecond time scale and the nanometer length scale, traditional shock characterization has been largely limited to post-mortem techniques, such as electron microscopy^18–20^. However, post-mortem analysis can merely infer the complex mechanistic history. Such deduction is particularly difficult when multiple mechanisms activate and deactivate over such small scales. Simulations have provided valuable insight into this process^21^ but cannot replace in situ experimentation. Most common in situ techniques, such as laser interferometry, lack the spatial resolution necessary for detailing transient microstructures. However, researchers have just begun to harness high-speed X-ray diffraction to detail an experimental shock response in real-time^22,23^. Over just the last five years, this approach has resolved a long-standing controversy over the phase transformations of shocked graphite^24^, connected stacking faults to the plastic deformation of shocked Au^25^, tracked the twins and slip in shocked Mg^23,26^, demonstrated the pressure dependence of slip and twining for Ta^8^, and elucidated the phase transformation of BCC Fe^27^. Moreover, researchers have just begun to harness high-speed electron diffraction for even finer spatial resolution^28–31^.

While high-speed diffraction can theoretically track nanoscale deformations in real-time, the traditional methods for interpreting diffractograms are unfortunately lacking^32,33^. For example, numerous methods for characterizing defects from the widths of diffractogram peaks often yield conflicting results^34–36^. To resolve such uncertainties, researchers have recently generated virtual diffractograms from atomistic simulations^36–38^. By comparing the ground truths of the atomistic simulations to interpretations of the corresponding virtual diffractograms, they evaluated the efficacies of the interpretative methods themselves. These relatively inexpensive simulations are statistically robust, avoid measurement-induced damage, and can even match the time scale of a shock experiment^39^. In fact, several recent studies have accurately simulated diffractograms of shocked materials^40–43^.

Classical molecular dynamics simulations provide the ability to directly correlate atomic microstructures with their diffraction patterns during the various stages of shock loading. For the current work, we simulated the shock responses for single crystals of [110] Ta, [110] Fe, and [111] Cu during various stages of loading. These systems were chosen to feature three prominent modes of deformation in FCC and BCC metals: dislocation slip (stacking faults), deformation twining, and phase transformation. To facilitate the experimental, real-time tracking of these modes, we then produced virtual diffractograms from selected volumes of each of the atomistic simulations. Specifically, we performed both powder X-ray diffraction (XRD) and selected area electron diffraction (SAED). The powder XRD effectively represented a large-wavelength, 1D line profile, and the SAED represented a small-wavelength, 2D diffraction pattern. Fingerprints of both types of diffractogram were identified and then correlated with the operating deformation modes. Especially when harnessed in concert with knowledge of the local pressures and plasticity contributors provided by atomistic simulations, these fingerprints have the potential to supercharge the quantitative interpretation of experimental diffractograms from shocked crystals.

## Results and discussion

### Deformation twinning in Ta

**Figure 1:**
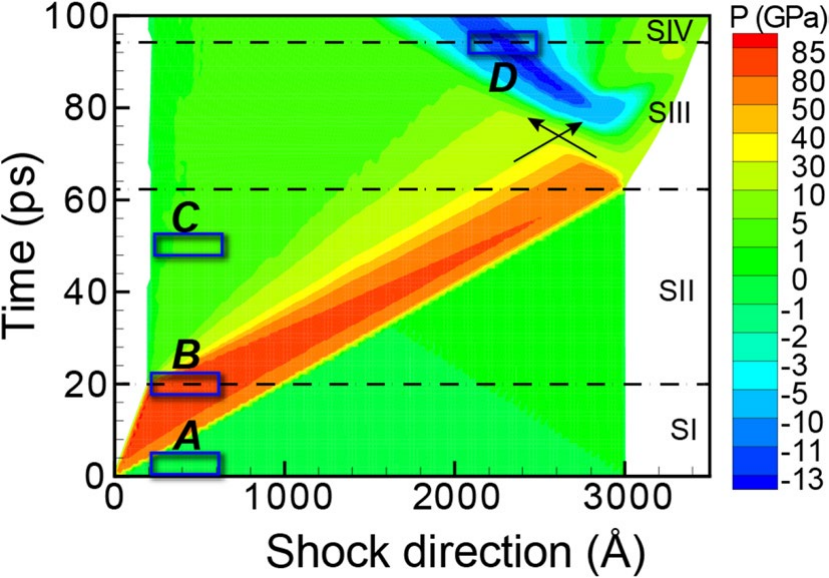
The temporal evolution of pressure for shocked Ta. The stages of shock are separated by dashed lines, and the region of triaxial tension is indicated by black arrows. Blue rectangles, labeled A–D, mark the regions examined in Fig. 2 and Table S1.

When subjected to shock loading, Ta predominantly twins due to the compressive wave and then quickly detwins due to the release wave. Hence, twinning in Ta represents a transient deformation mode that can only be experimentally observed via a high-speed, in situ method. For comparison with such experiments, we simulated shock via molecular dynamics for a 300-nm bar of Ta oriented along [110]. Figure 1 presents the resulting pressure evolution for the full simulation domain as a function of both position and time. This pressure profile comprises four stages: stage I (SI) for the generation of compressive wave (i.e., elastic than plastic), stage II (SII) for the propagation of the compression wave and the generation/propagation of release wave, stage III (SIII) for the reflection/interaction of release waves and resulting nucleation of voids, and stage IV (SIV) for growth and coalescence of voids to initiate spall failure. As reflected by the color coding, the pressure varied between ∼ 87 GPa of compression behind the shock front and ∼ 13 GPa of tension at the spall plane.

From these atomistic simulations, we selected the 40-nm regions depicted by blue rectangles in Fig. 1 in order to evaluate the evolution of twinning throughout the shock loading. Specifically, these regions featured the Ta microstructure (a) at the initial state, (b) during shock compression, (c) after shock release, and (d) under tensile pressure. Next, we quantified the twin fraction and produced both virtual SAED and virtual XRD for each of these selected regions. By correlating the ground truths of the twin densities with the changes in the diffractograms, we identified the fingerprints of shock-induced twinning for both 2D and 1D diffractograms. For reference, the twin densities and other defect densities are summarized in Table S1 provided in Supplementary Information.

**Figure 2:**
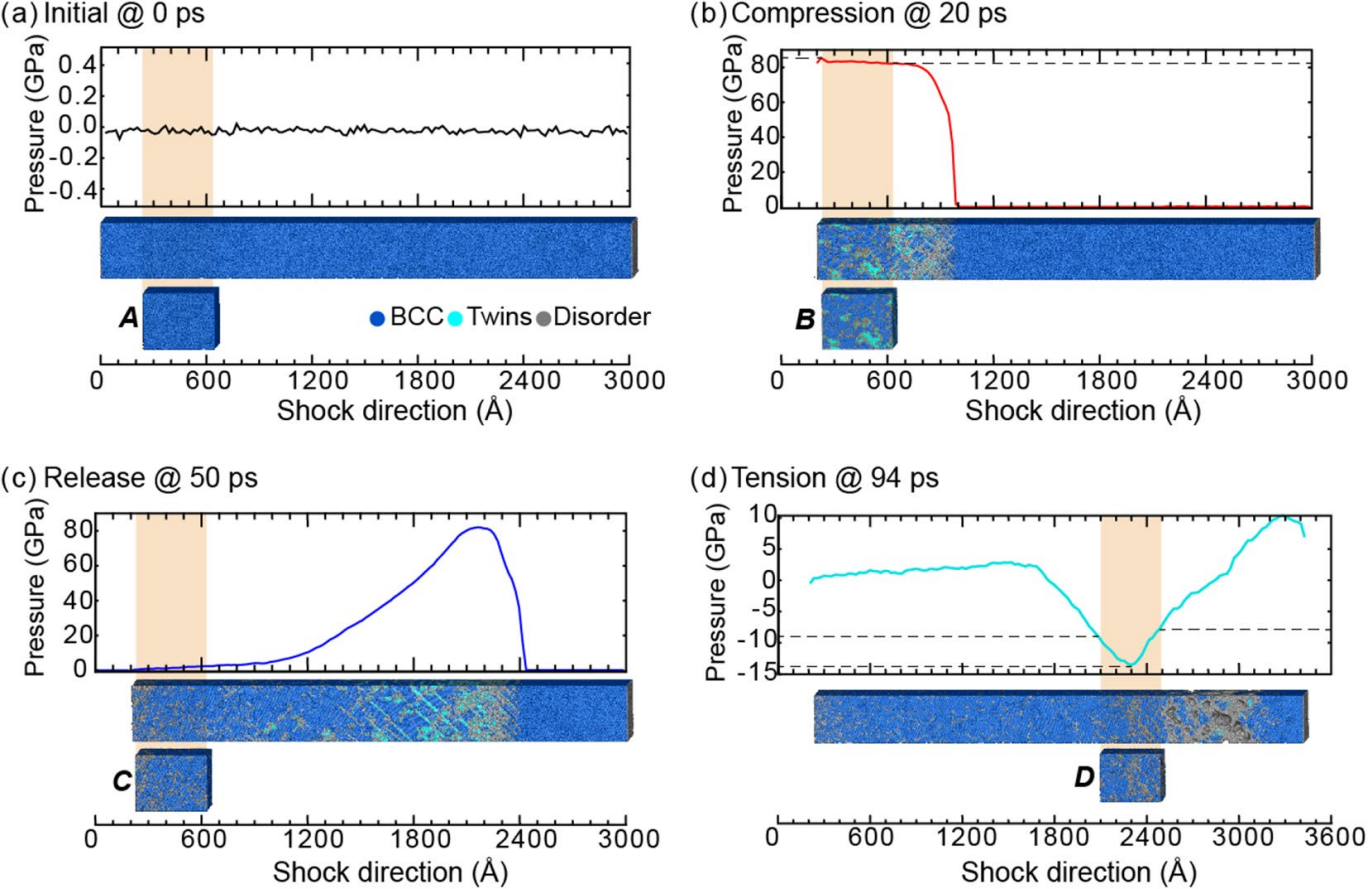
The pressure distribution and microstructure for select time steps of shocked Ta. Cropped regions to be assessed with virtual diffraction are highlighted in tan. The cropped regions, labeled A–D, correspond to the blue rectangles in Fig. 1.

**Figure 3:**
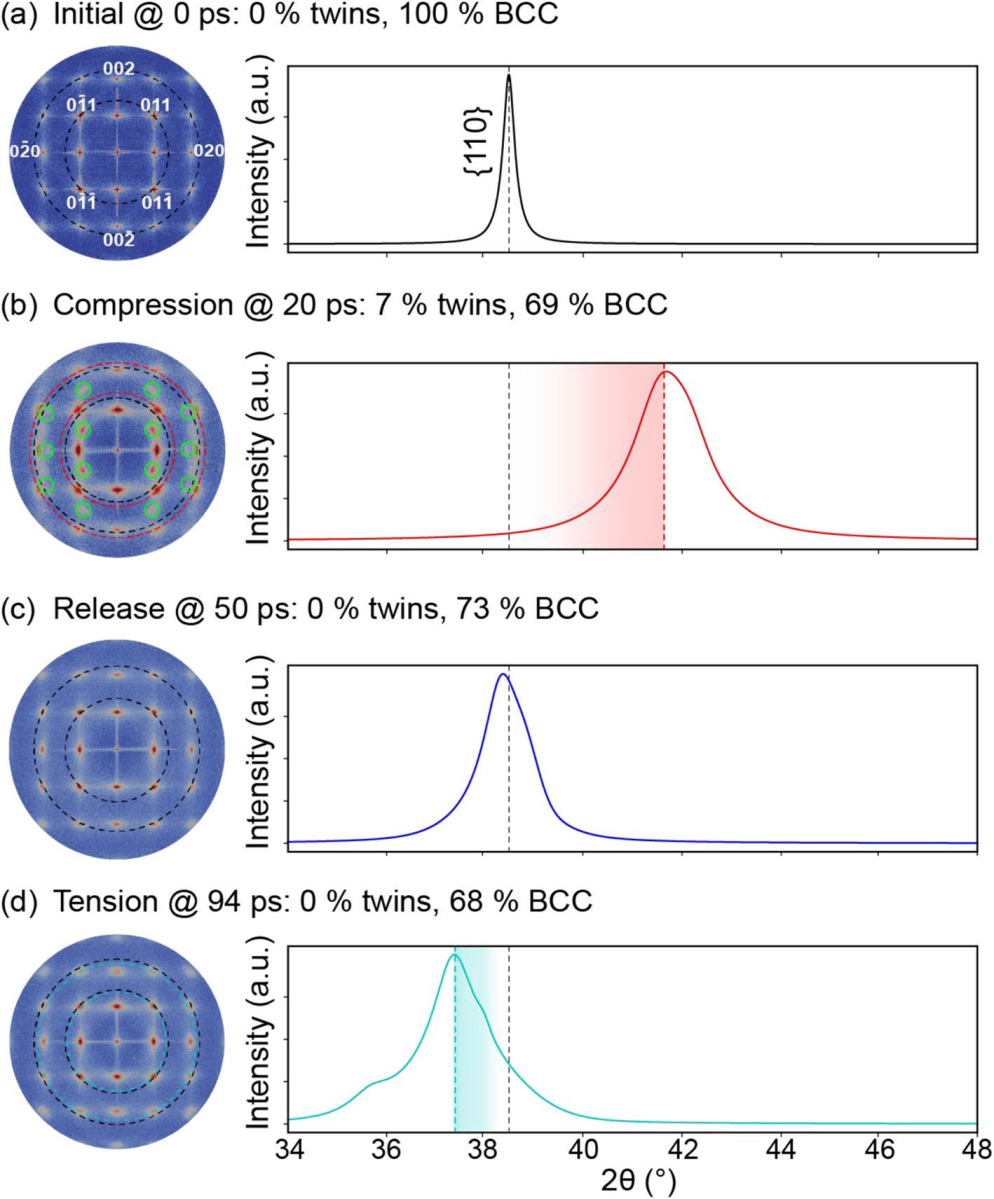
Virtual diffraction for BCC Ta shocked along [110] at (**a**) 0 ps, (**b**) 20 ps, (**c**) 50 ps, and (**d**) 94 ps after impact. Each SAED corresponds to a [100] zone axis, a 1.25-Å^−1^ radius, and 200-keV electron irradiation. Each XRD represents powder diffraction for Cu-Kα X-rays. For association with diffraction signatures, discrete quantifications of twins and phases are provided in the subfigure labels. For reference, new SAED spots are indicated with solid circles, and the locations of SAED spots and the XRD peak are indicated by dotted lines color-coded by time step. The SAED spots for low-order planes are indexed. SAED patterns are plotted using Paraview^66^, version 5.7 from https://www.paraview.org/.

Initially, the Ta sample had a BCC microstructure (Fig. 2a), which produced sharp spots in the SAED pattern and a sharp peak in the XRD profile (Fig. 3a). For reference, the equilibrium positions of some SAED spots and the XRD peak are denoted by dotted black lines across all sub-figures in Fig. 3. At the moment of impact, the compression wave materialized and quickly increased the pressure in Ta (Fig. 2b). Correspondingly, the SAED spots shifted outward from the dotted black circles to the dotted red circles (see Fig. 3b). Interestingly, the SAED spots lying on the ordinate or abscissa broadened elliptically, whereas the others deformed more circularly. Structural analysis indicated that the plastic compressive wave generated twins at the shock front. For example, the twins accounted for 7% of the volume for the 20-ps slice. The SAED reflected this twinning in two prominent fingerprints: (i) the emergence of (112) mirror-plane spots (marked with solid green circles in Fig. 3b) and (ii) splitting of the original spots. These mirror-plane spots correspond to the primary twin plane in BCC metals. The intensities of the new spots were lower than the intensities of the preexisting spots because the twins corresponded to only 7% of the volume, as compared to the 69% for the BCC structure. The emergence of new spots in the SAED pattern under compression agrees with the experimental observation of new spots representing twinning in *in-situ* Laue XRD of Ta^8^. For the 1D XRD line profile, the shock compression induced an upward peak shift from the dotted black line to the dotted red line (see Fig. 3b) as well as a peak broadening. The peak shift was due to the average pressure of ~ 83 GPa in the analyzed section, and the peak broadening was largely due to the densities (volume fractions) and distributions of dislocations and twins (as tabulated in Table S1). Researchers have often attempted to decouple the effects of the various broadening sources for 1D XRD line profiles, but no solution has been universally accepted^35,36^.

By 50 ps, the propagation of the newly generated release wave had unloaded the material to zero pressure in the analyzed slice (Fig. 2c). Correspondingly, the SAED spots and the XRD peak shifted near to their original locations (dotted black circles/line in Fig. 3c). Consistent with the aforementioned literature, the structural analysis indicated that this release wave initiated a detwinning process. For the 50-ps slice, no deformation twins were observed. Consequently, the mirror-plane spots disappeared from the SAED pattern, and the XRD peak narrowed. However, some broadening of the SAED spots and of the XRD peak remained because of dislocations and other defects (Table S1 in Supplementary Information).

By 94 ps, the compressive wave had reached the end of the sample, reflected as a rarefaction wave, and interacted with the tail of the pressure wave to maximize tension at 13.7 GPa. Correspondingly, the SAED spots shifted inward from the black circles to the cyan circles, and the XRD peak shifted to a lower 2θ value (Fig. 3d). This tensile pressure increased the dislocation density (Table S1 in Supplementary Information) and correspondingly broadened the SAED spots and the XRD peak. Because the analyzed slice contained a range of pressures (i.e., 10 to 13 GPa of tension) (Fig. 2d), the peak broadened heterogeneously (Fig. 3d). If the pressure had been homogeneous across the analyzed slice, the XRD peak would have uniformly shifted, and we would have fit this peak with a single pseudo-Voigt curve rather than with several (see Note 2 of the Supplementary Information for curve fits). Overall, our simulation results validated the formation of mirror-plane spots and the splitting of preexisting spots as the fingerprints of deformation twinning.

### Phase transformations and twinning in Fe

**Figure 4:**
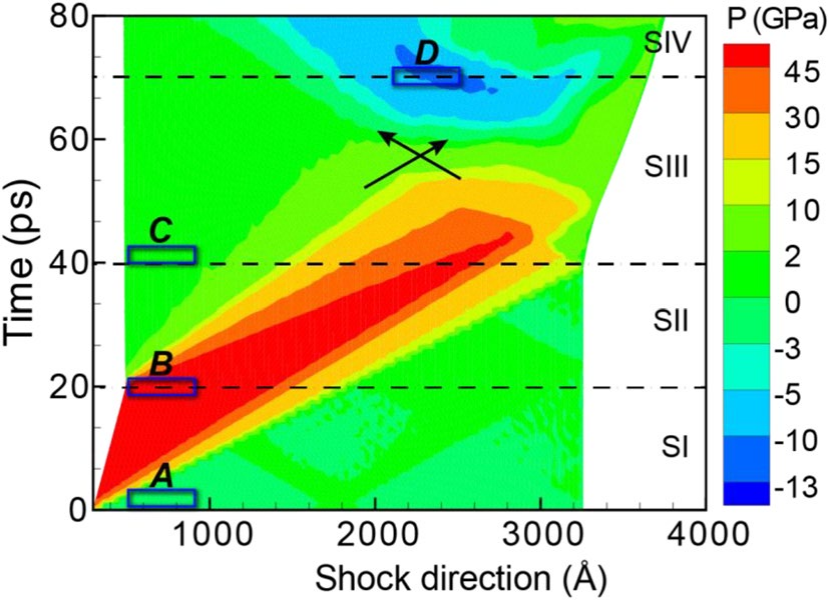
The temporal evolution of pressure for shocked Fe. The stages of shock are separated by dashed lines, and the region of triaxial tension is indicated by black arrows. Blue rectangles, labeled A–D, mark the regions examined in Fig. 5 and Table S2.

After identifying the characteristic signatures for twins in the diffractograms of shocked Ta, we turned to BCC Fe shocked along [110] to focus on phase transformations. Recall that shock typically induces only twins in Ta but both twins and phase transformations in Fe. In shocked Fe, a compressive wave induces an α (BCC) → ϵ (HCP) phase transformation, and the release wave quickly reverses this transformation to generate twins in the BCC microstructure. Having first understood the effect of twins on the diffractograms of shocked Ta, we isolated the effects of phase transformations in shocked Fe. Figure 4 presents the pressure profile for our atomistic shock simulation. As reflected by the color coding, the pressure varied between ∼ 54 GPa of compression and ∼ 11 GPa of tension.

As depicted by the blue rectangles in Fig. 4, we selected 40-nm regions at specific times to feature the microstructural evolutions of the shocked Fe. These regions featured Fe (a) in the initial BCC configuration, (b) after the HCP phase transformation, (c) with twinning after shock release, and (d) under tensile pressure. We then characterized both the twin and phase fractions for use as ground truths (see Table S2 in Supplementary Information). As with Ta, we also produced virtual 2D SAED patterns and 1D XRD line profiles for comparison with the microstructural ground truths in order to detail the fingerprints of these defects in the diffractograms.

**Figure 5:**
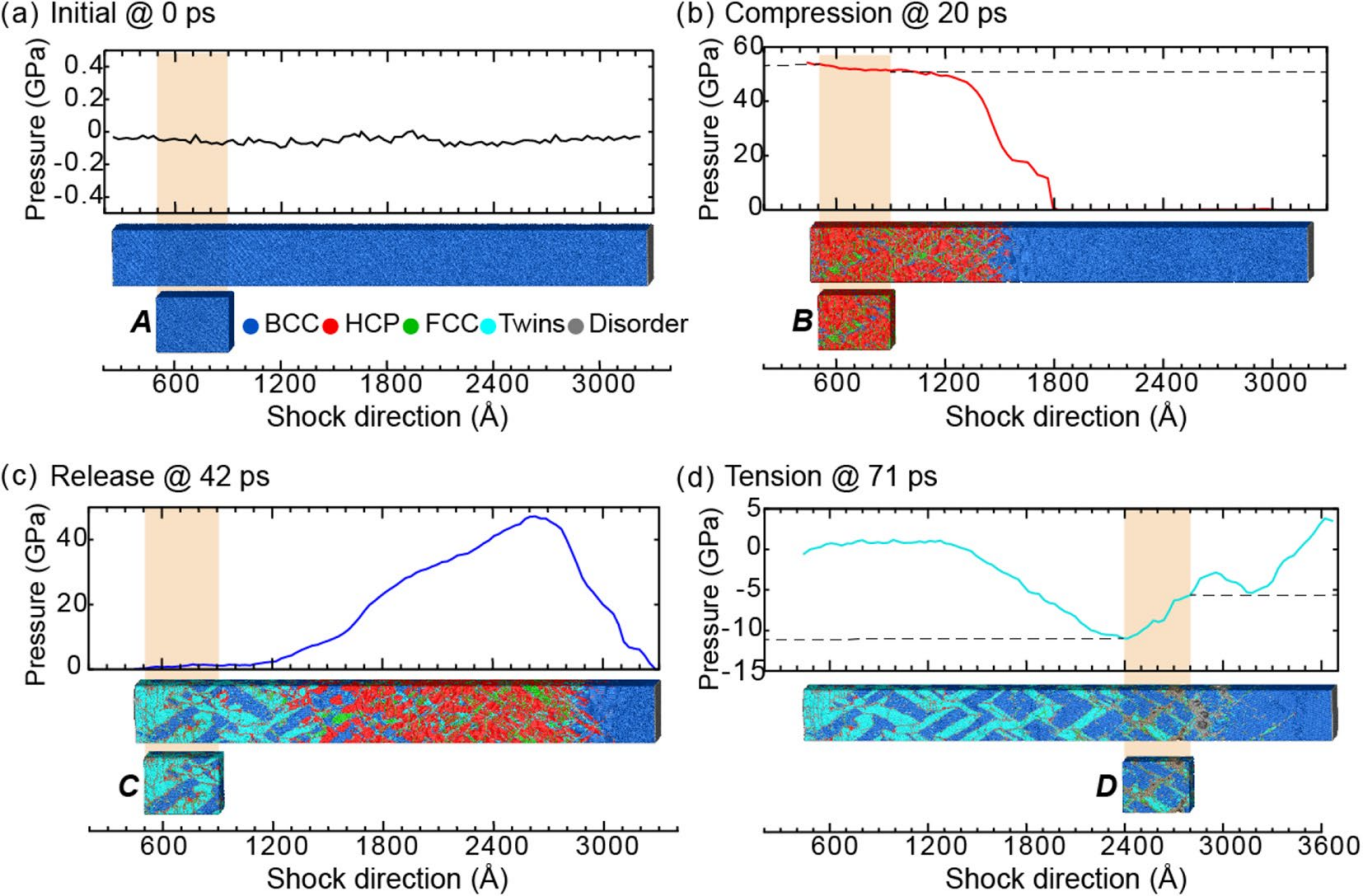
The pressure distribution and microstructure for select time steps of shocked Fe. Cropped regions to be assessed with virtual diffraction are highlighted in tan. The cropped regions, labeled A–D, correspond to the blue rectangles in Fig. 4.

**Figure 6:**
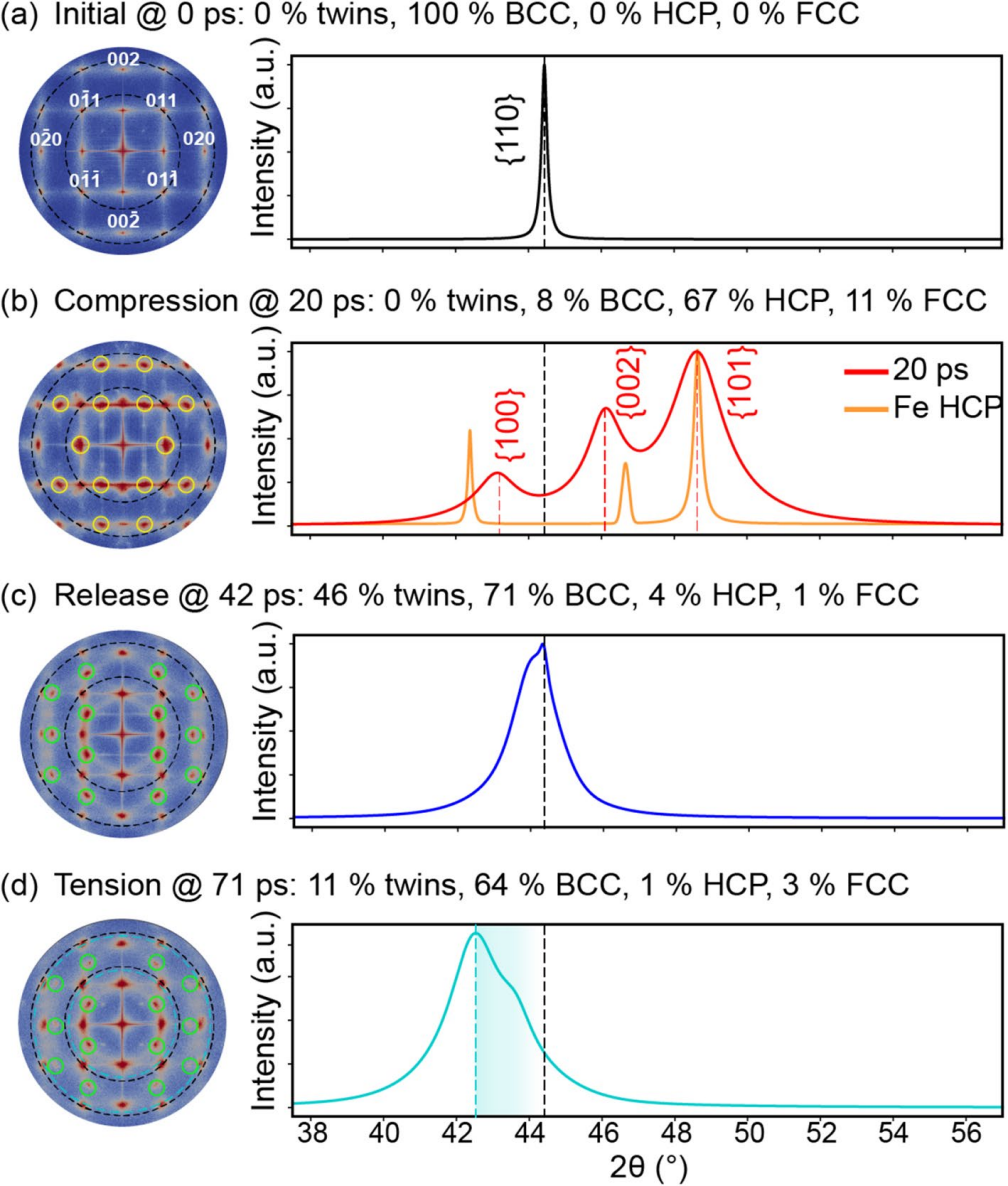
Virtual diffraction for BCC Fe shocked along [110] at (**a**) 0 ps, (**b**) 20 ps, (**c**) 42 ps, and (**d**) 71 ps after impact. Each SAED corresponds to a [100] zone axis, a 1.25-Å^−1^ radius, and 200-keV electron irradiation. Each XRD represents powder diffraction for Cu-Kα X-rays. For association with diffraction signatures, discrete quantifications of twins and phases are provided in the subfigure labels. For reference, new SAED spots are indicated with solid circles, and the locations of SAED spots and the XRD peak are indicated by dotted lines color-coded by time step. The SAED spots for low-order planes are indexed. SAED patterns are plotted using Paraview^66^, version 5.7 from https://www.paraview.org/.

The SAED/XRD for the initial BCC Fe microstructure (Fig. 5a) had sharp spots/peaks (Fig. 6a). The shock compression of Fe at a pressure of 54.1-GPa induced a BCC-HCP phase transformation. For the 20-ps slice (Fig. 5b), the BCC spots in the SAED marginally shifted outward but were accompanied by a new set of HCP spots (Fig. 6b). The high intensities of these HCP spots corresponded to the high-volume fraction of the HCP phase (i.e., 67% HCP vs. 8% BCC, as tabulated in Table S2). The XRD line profile evolved from a single peak corresponding to the BCC phase to three peaks corresponding to the HCP phase. Discrepancies between the reference profile (orange curve in Fig. 6b) and the 20-ps profile (red curve in Fig. 6b) were due to the distribution of pressure and non-HCP phases (Table S2 in Supplementary Information). For example, the peak at 46.0° likely resulted from a superposition of the middle HCP peak and the original BCC peak (shifted due to compression). By 42 ps, the release wave had formed, relaxed the assessed slice, and reversed the phase transformation from the high-pressure HCP phase back to the low-pressure BCC phase (Fig. 5c). Correspondingly, the HCP spots/peaks disappeared from the SAED/XRD (Fig. 6c).

The release wave not only reversed the phase transformation but left behind a significant twin fraction (e.g., 46%) in the 42-ps microstructure. Although these twins were caused by a release wave in Fe rather than by a compressive wave in Ta, their diffraction signatures were similar. The twins again split preexisting spots in the SAED, produced (112) mirror-plane spots in the SAED, and broadened the XRD peak (Fig. 6c). By 71 ps, the compressive wave had transformed into a tensile rarefaction wave that reduced the twin fraction from 46 to 11% in the analyzed slice. Predictably, this tensile pressure, which maximized at 11.1 GPa, shifted the SAED spots inward and the XRD peak downward to a lower 2θ value (Fig. 6d). As in the case of Ta, the heterogeneous shape of the XRD peak was due to a distribution of tensile pressures, as shown by the range from 5 to 10 GPa of tension for the cropped region in Fig. 5d. Overall, these Fe results demonstrated how dislocations, phase transformations and twins collectively evolve both 1D and 2D diffractograms. By harnessing the fingerprints identified in this investigation and knowledge of local pressures and plasticity contributors from atomic-scale simulations, researchers can decouple these superimposed deformation modes when analyzing experimental diffractograms^27^.

### Stacking faults in Cu

**Figure 7:**
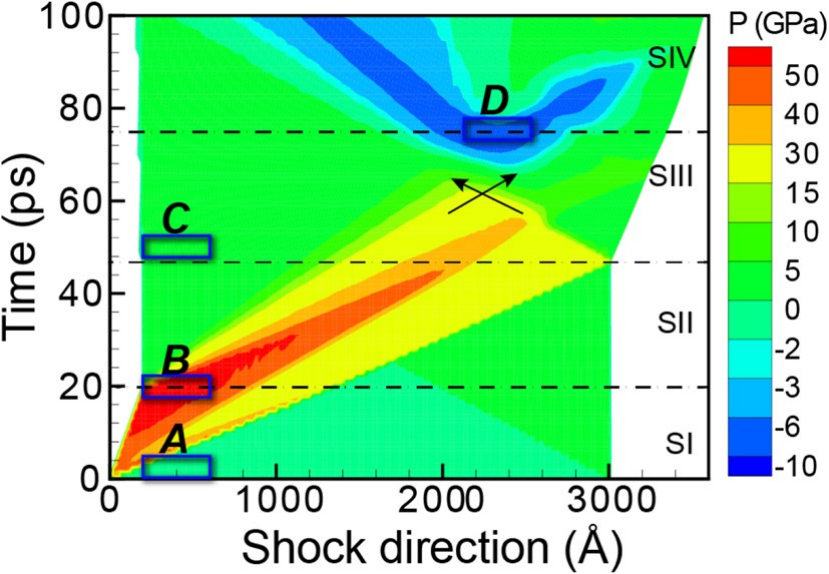
The temporal evolution of pressure for shocked Cu. The stages of shock are separated by dashed lines, and the region of triaxial tension is indicated by black arrows. Blue rectangles, labeled A–D, mark the regions examined in Fig. 8 and Table S3.

After examining Ta and Fe to fingerprint deformation twins and phase transformations in diffractograms, we examined Cu to focus on stacking faults. When shocked specifically along [111], Cu predominantly forms stacking faults. Note that other shock directions would feature twins because Cu has a relatively low stacking-fault energy. For our atomistic shock simulation, Fig. 7 shows how the pressure varied between ∼ 53 GPa of compression and ∼ 10 GPa of tension.

**Figure 8:**
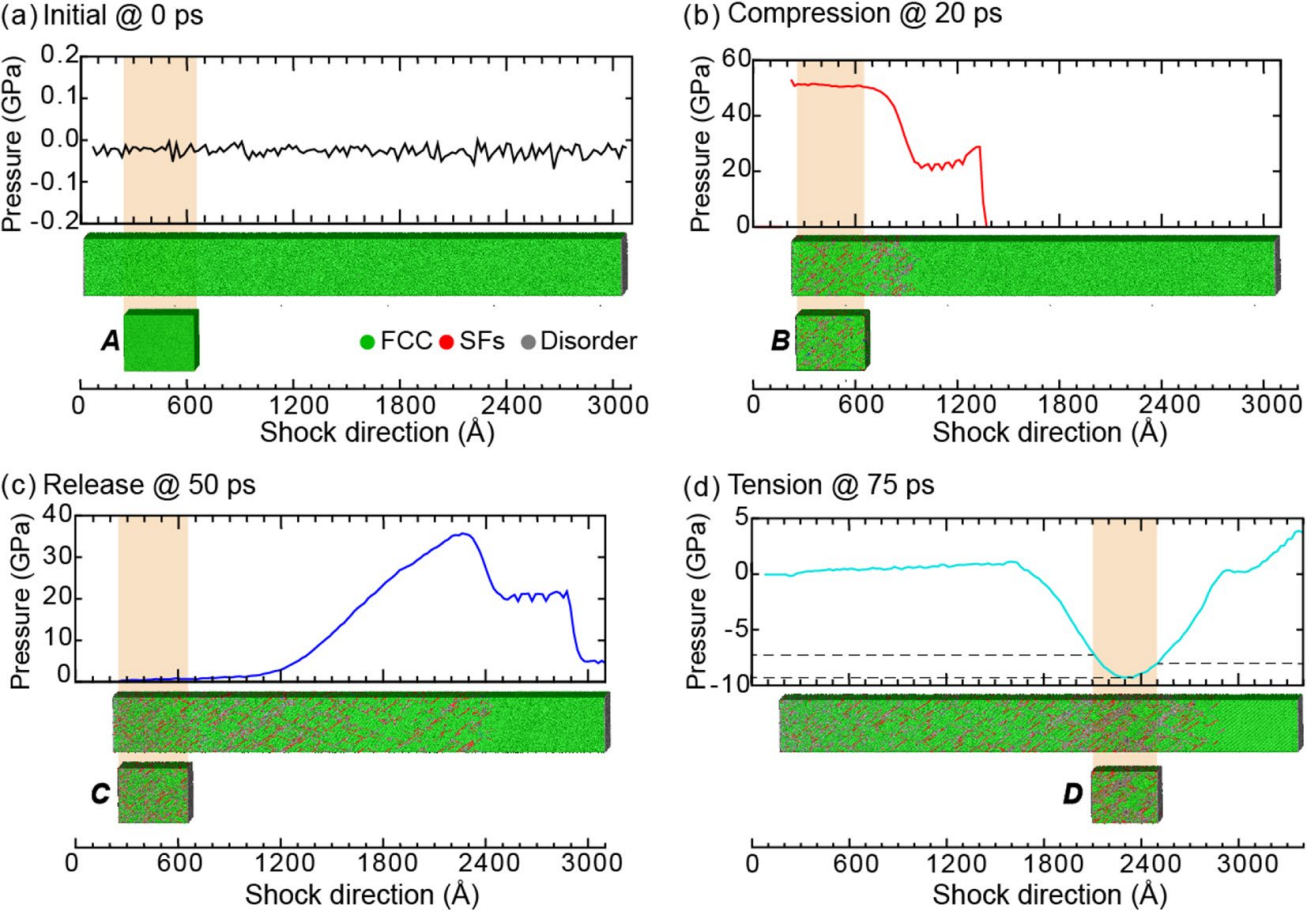
The pressure distribution and microstructure for select time steps of shocked Cu. Cropped regions to be assessed with virtual diffraction are highlighted in tan. The cropped regions, labeled A–D, correspond to the blue rectangles in Fig. 7.

For the 40-nm regions indicated by blue rectangles in Fig. 7, we characterized the microstructural defects (see Table S3 in Supplementary Information) and produced virtual diffractograms (Fig. 8). These regions corresponded to the Cu (a) in the initial FCC microstructure, (b) during shock compression, (c) after unloading, and (d) under tension. Interestingly, the stacking faults induced by the shock compression largely persisted in our simulations even after the release wave and the rarefaction wave passed. In contrast, recall that the release wave substantially reduced the twin fractions in Ta and the HCP phase in Fe. Therefore, stacking faults were not a transient mode of deformation in Cu. However, being able to observe the evolution of the stacking faults during the short time scale of a shock loading and identify their contributions to peak shifts and peak broadening is still valuable. The signatures of these stacking faults would likely be consistent regardless of their transience just as the signatures of the twins remained constant for the different conditions in the Ta and Fe discussed earlier.

**Figure 9:**
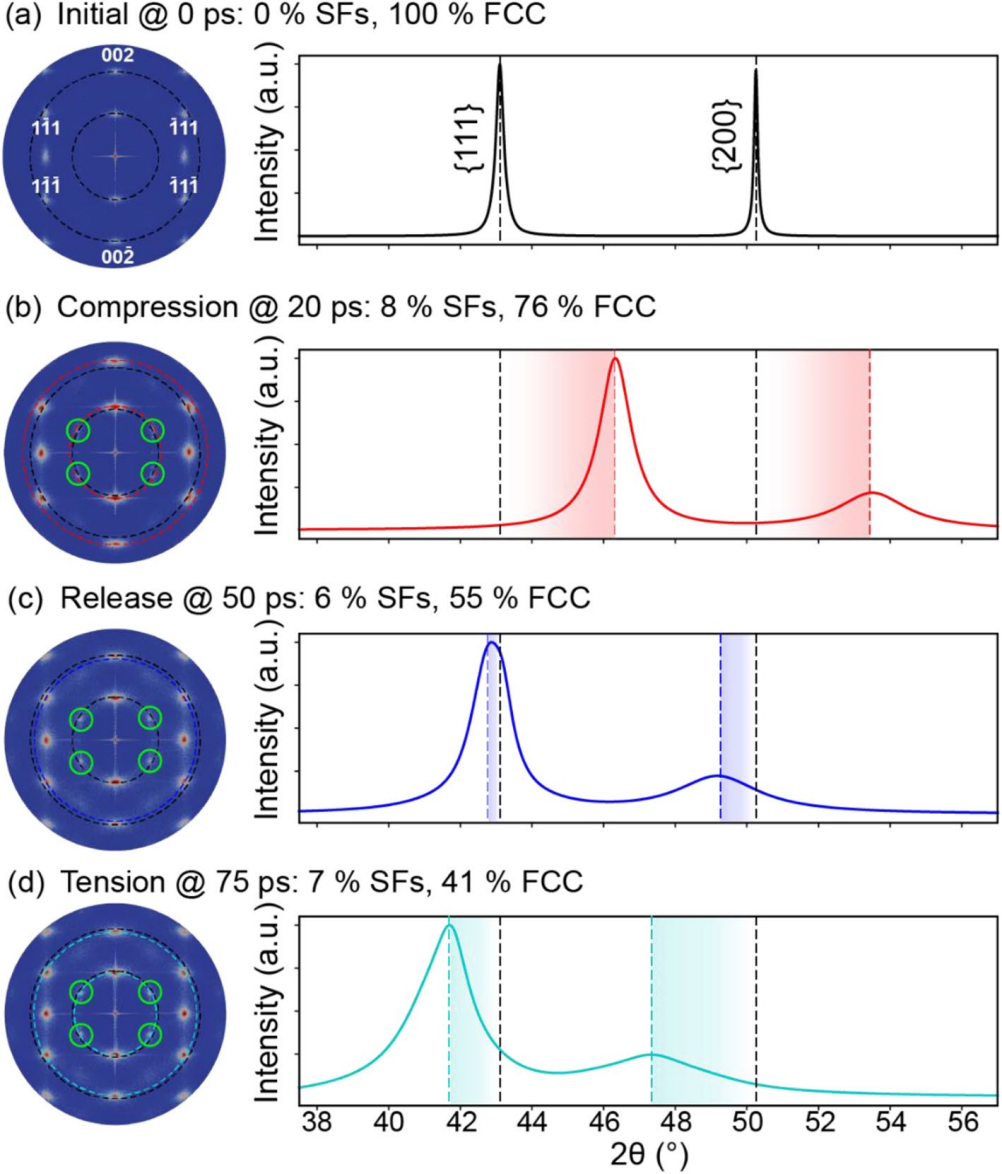
Virtual diffraction for FCC Cu shocked along [111] at (**a**) 0 ps, (**b**) 20 ps, (**c**) 50 ps, and (**d**) 75 ps after impact. Each SAED corresponds to a [110] zone axis, a 1.25-Å^−1^ radius, and 200-keV electron irradiation. Each XRD represents powder diffraction for Cu-Kα X-rays. For association with diffraction signatures, discrete quantifications of stacking faults (SFs) and phases are provided in the subfigure labels. For reference, new SAED spots are indicated with solid circles, and the locations of SAED spots and XRD peaks are indicated by dotted lines color-coded by time step. The SAED spots for low-order planes are indexed. SAED patterns are plotted using Paraview^66^, version 5.7 from https://www.paraview.org/.

As in the cases of Ta and Fe, the initial microstructure of Cu was 100% crystalline (Fig. 8a) and thus produced sharp spots/peaks in the SAED/XRD (Fig. 9a). Of course, the FCC crystal structure of the Cu and the corresponding diffractograms qualitatively differed from those of the BCC Ta/Fe. For example, in a similar range of 2θ, the XRD displayed two peaks rather than one because of differences in the extinction rules. By 20 ps, the Cu experienced a maximum compressive pressure of 53.0 GPa (Fig. 8b) and the generation of stacking faults (as evidenced by HCP crystal structure). Correspondingly, the SAED spots shifted outward, and the XRD peaks shifted upward to a higher 2θ value (Fig. 9b). Note that these stacking faults not only broadened but also shifted the spots/peaks^25^. Excluding the role of pressure, the presence of stacking faults in the FCC phase would shift {111} and {200} peaks to higher and lower 2θ values, respectively^44^. For example, at 20 ps, the 8% of stacking faults increased the compression-induced, upward shift of the {111} XRD peak by 3.4° but lessened the upward shift of the {200} peak by 3.2° (see Fig. S1 for a full assessment of the peak shifts due to stacking faults). Therefore, the combination of a heterogeneous shift and a broadening of the XRD peaks could be used to fingerprint stacking faults in 1D diffractograms. The aforementioned methods for decoupling the effects of various broadening sources in XRD could be used to quantify the relative contributions if the model parameters were optimized.

By 50 ps, the release wave had removed the pressure from the analyzed slice, healed some Shockley partial dislocations, and reduced the stacking-fault volume fraction from 8 to 6% (Fig. 8c and Table S3 in Supplementary Information). Because of the stress-free condition, the radial positions of the SAED spots almost relaxed back to their equilibrium positions (Fig. 9c). However, the residual 6% of stacking faults retained the shift of the XRD peaks to a lower 2θ value, especially for the {200} peak. In contrast to the shock of Ta or Fe, the elastic compressive wave was much faster than the trailing plastic compressive wave in Cu. The elastic compressive wave reflected off the end of the sample at 47 ps and interacted with the entire plastic wave before surpassing it at 63 ps, as shown in Fig. 7. The plastic wave finally reflected off the end of the sample at 65 ps and increased the tensile pressure to 9.9 GPa at 75 ps (Fig. 8d). Interestingly, this tensile pressure slightly increased the fraction of stacking faults from 6 to 7%. As in the cases of Ta and Fe, the tensile pressure shifted the SAED spots inward, and the XRD peaks to a lower 2θ value (Fig. 9d). As evidenced by the diffusion of the FCC spots and the presence of new spots in the SAED, the stacking faults largely persisted with a volume fraction of 7%. Note that if twins were present (as is common for other shock directions of Cu because of its low stacking fault energy), the twins could have also been distinguished from stacking faults via SAED. While both twins and stacking faults induce the formation of new spots, only twins cause the splitting of spots.

## Conclusions

We systematically linked salient deformation modes for shocked metals to two types of diffractograms by atomistically simulating shocked microstructures, 1D XRD line profiles (large-wavelength), and 2D SAED diffraction patterns (small-wavelength). For the Ta system, the results demonstrated that deformation twins induce broadening of peaks in 1D diffractograms and the splitting of spots in 2D diffractograms. Because peak broadening can be induced by numerous types of defects, further knowledge, such as the distribution of local pressures and plasticity contributors, must be incorporated in order to quantify the contributions from dislocations and twins in line profiles. In contrast, the spot splitting in 2D diffractograms serves as a twinning fingerprint. For the Fe system, the results demonstrated that phase transformations manifest in both 1D and 2D diffractograms as new peaks and spots, respectively. The results also demonstrated that the simultaneous activation of multiple deformation modes can be identified via diffractograms. In this case, twins nucleated and affected the diffractograms in similar fashion as with the aforementioned Ta despite differences in loading style. For the Cu system, the results demonstrated that stacking faults also affect both types of diffractograms. The HCP structure of the stacking faults induced the formation of new spots in the 2D diffractogram and the broadening and shift of the peak in the 1D diffraction pattern.

Overall, this study demonstrates how generating virtual diffractograms from atomistic datasets can facilitate the fingerprinting of deformation modes that operate during the complex wave propagations and interactions during shock loading. Specifically, these results detail how transient modes of deformation, such as dislocation slip, deformation twinning, and phase transformation, induce the generations, shifts, splitting, and broadening of peaks/spots in 1D/2D diffractograms. This capability will facilitate the understanding and optimization of shock responses by empowering the real-time, nanoscale characterization of shocked materials via high-speed diffraction. With such methods, experimentalists will be able to observe and understand the complex microstructural evolutions inaccessible to post-mortem characterizations and continuum measurements.

## Methods

### Molecular dynamics simulations

We performed atomistic shock simulations for Ta, Fe, and Cu via the Large-scale Atomic/Molecular Massively Parallel Simulator (LAMMPS)^45^ by Sandia National Laboratories. For each material, we constructed a representative volume (30 nm × 30 nm × 300 nm) oriented such that a shock wave would travel along [110] for a BCC system (i.e., Ta or Fe) or along [111] for the FCC system (i.e., Cu).

Embedded-atom-method (EAM) potentials for Ta^46^, Fe^47^, and Cu^48^ represented the interatomic interactions. These potentials reproduced the Hugoniot response observed in experimentation^49–51^. The Ta potential reproduced the twinning predicted by density functional theory^8,46,50,51^. The Fe potential reproduced the BCC-HCP phase transformation close to the experimental pressure of ∼ 13 GPa^47,52–54^. The Cu potential has been used to study the shock and spall response for the range of microstructure and loading conditions^49,55–57^.

We used a Nośe-Hoover ensemble (NPT) to equilibrate the microstructures at 300 K and 0 GPa. We shocked each system by driving a 3-nm piston into the metal sample at a constant velocity of 1 km/s for a pulse of 20 ps. For each simulation, we computed the temporal evolution of pressure via spatial binning along the shock direction. We also characterized the phase compositions, dislocation densities, twin densities, and stacking-fault densities^58–62^. Twins were identified by performing polyhedral template matching (PTM)^63^ on atoms with a similar structure type to identify Euler angles corresponding to mismatches greater than 10° angle.

### Virtual diffraction

For select, 40-nm regions of the shocked microstructures, we non-periodically simulated both 2D SAED diffraction patterns and 1D powder XRD line profiles via the LAMMPS user-diffraction package^64,65^. This package first constructed diffraction vectors corresponding to each point on a mesh in a selected volume of reciprocal space. We choose a mesh spacing of 0.0015 Å^−1^ for XRD and 0.0010 Å^−1^ for SAED. We selected the volume by setting the maximum diffraction vector to 1.25 Å^−1^ for SAED and limiting 2θ from 0° to 60° for XRD. The kinematic diffraction intensity was computed for each of these vectors via the irradiation wavelengths, the atomic scattering factors, and a polarization factor (for XRD only). For the wavelengths, we selected 0.0251 Å to model 200-keV electrons for SAED and 1.54 Å to simulate Cu Kα for conventional powder XRD. Each of the scattering factors was parameterized specifically for the simulated material and the type of diffraction. Finally, the diffraction patterns were generated from the grid of diffraction intensities. For SAED, the grid was sliced according to an Ewald sphere with a radius inverse of the wavelength, a position corresponding to desired zone axis, and a thickness of 0.005 Å^−1^. Then, the intensities within that slice were projected in the direction of the zone axis to create a SAED pattern within Paraview^66^. Alternatively, for powder XRD, an Ewald sphere was rotated around the origin of reciprocal space. The line profile was constructed by binning the resulting intensities according to their diffraction angle and then smoothed by fitting with pseudo-Voigt curves^67^ via SciPy^68^. Note 2 of the Supplementary Information provides the parameters of these XRD fits. Because of the SAED projection and the spatial averaging in the XRD, the relative contribution of the defected regions to the overall diffractogram could change in prominence if the simulation volume were altered. However, the nature of their contributions would not. Therefore, the diffractogram fingerprints identified by our work would still be relevant when assessing larger material volumes in experiments.

## Supplementary information

Supplementary Information.

## Data Availability

The datasets generated during and/or analyzed during the current study are available from the corresponding author on reasonable request.

## Code availability

All simulations were executed using open-source software LAMMPS (https://lammps.sandia.gov/).
