# Supplementary material for: Fingerprinting shock-induced deformations via diffraction
Source: Sci Rep. 2021 May 10;11:9872. doi: 10.1038/s41598-021-88908-y (PMC8111029; doi:10.1038/s41598-021-88908-y)
Supplement: Supplementary file 1 — Supplementary Information. [file 41598_2021_88908_MOESM1_ESM.pdf]

Supplementary Information for

Fingerprinting Shock-Induced Deformations via Diffraction

Avanish Mishra,[1,2] Cody Kunka,[3] Marco J. Echeverria,[1,] Rémi Dingreville,[3,*] and Avinash M. Dongare[1,2,†]

[1] Department of Materials Science and Engineering, University of Connecticut, Storrs, CT 06269, USA

[2] Institute of Materials Science, University of Connecticut, Storrs, CT 06269, USA

[3] Center for Integrated Nanotechnologies, Sandia National Laboratories, Albuquerque, NM87123, USA

[*] Corresponding author, electronic mail: rdingre@sandia.gov
[†] Corresponding author, electronic mail: dongare@uconn.edu

Note 1: Microstructural Evolution

Table S1, S2, and S3 detail the average pressure, the volume fraction of twins (or stacking faults), and dislocations density for 40 nm cropped regions for Ta, Fe, and Cu at different timesteps, respectively. Unlike the cases of Ta and Fe, Cu predominantly experiences stacking faults for the considered shock direction.

Table S1: Average pressure, twin fraction, and dislocation density for the cropped regions of shocked Ta.

Time (ps)	Pressure (GPa)	Twins (%)	Dislocation density (nm^{-2})		
			1/2<111>	<100>	<110>
0	0	0	0	0	0
20	83	7	2.12	0.35	0.04
50	1	0	1.05	0.16	0.01
94	-11	0	1.01	0.09	0.01

Table S2: Average pressure, phase and twin fractions, and dislocations density for the cropped regions of shocked Fe.

time (ps)	Pressure (GPa)	Phases (%)				Twins (%)	Dislocation density (nm^{-2})		
		BCC	HCP	FCC	Disorder		1/2<111>	<100>	<110>
0	0	100	0	0	0	0	0	0	0
20	52	8	67	11	14	0	0.05	0.01	0.0
42	1	71	4	1	24	46	0.53	0.07	0.01
71	-8	64	1	3	32	11	0.24	0.02	0.01

Table S3: Average pressure, stacking faults (SFs) fraction, and dislocations density for the cropped regions of shocked Cu.

Time (ps)	Pressure (GPa)	SFs (%)	Dislocation density (nm^{-2})				
			Perfect	Shockley	Stair	Hirth	Frank
0	0	0	0	0	0	0	0
20	51	8	0.21	4.09	0.31	0.19	0.04
50	0.5	6	0.24	2.83	0.32	0.14	0.05
75	-9	7	0.05	1.72	0.22	0.07	0.01

To validate peak shift and broadening due to stacking faults (SFs) in FCC metals, we generated XRD patterns for full microstructure, FCC atoms, SFs, and dislocations under compression at 20 ps, as shown in Figure S1. The XRD line profile for full microstructure and FCC atoms are shown by red and green colors, and the shaded region highlights the deviation between them. Figure S1 shows that excluding the stacking fault, XRD peaks for only FCC atoms (green curve) show the shift of the second peak to a higher diffraction angle compared full microstructure peak. The XRD for full microstructure (red curve) shows that the {111} peak is shifted towards a higher 2θ value compared to only FCC atoms line-profile (green curve), whereas the {200} peak is shifted towards lower 2θ value, as indicated by arrows. Moreover, the XRD profile for stacking faults shows dominated broadening to the right of the first XRD peaks, which suggests that in the presence of SFs, {111} and {200} peaks would shift to the right and left, respectively. These observations confirm that stacking faults in FCC metals would shift peaks originating from different {*hkl}* planes distinctively. The peak broadening due to dislocations is symmetrically distributed in the given 2θ range.

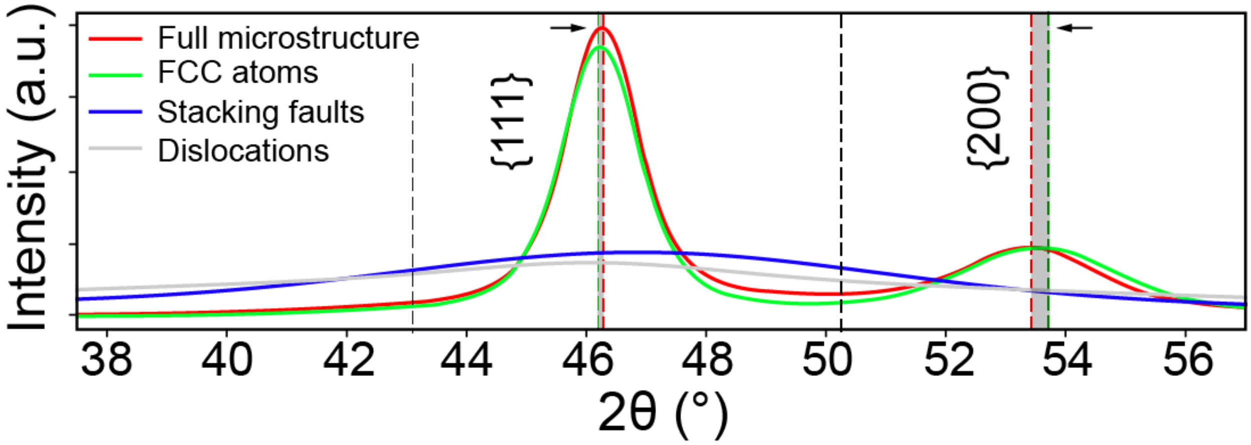

Figure S1: XRD for full microstructure, only FCC atoms, stacking faults, and dislocations of shocked Cu at 20 ps. The intensity of various XRD profiles should not be compared.

Note 2: XRD Fits

As mentioned in Section 1, we applied pseudo-Voigt curve fits to the raw XRD. Each pseudo-Voigt curve approximates the convolution of a Lorentzian (i.e., Cauchy) curve with a Gaussian curve. These curve fits are commonly used to assess XRD [1,2]. From these curve fits, we measured the two most common types of peak width because the widths indicate various types of defects, such as grain boundaries, twins, and stacking faults. The full-width-half-max width (FWHM), which indicates the width of a peak at half of its amplitude, is a popular measure for experimentalists because noise-covered peak tails do not need to be discerned. In contrast, theorists prefer the integral-breadth width (IB), which corresponds to the width of a rectangle that has the same amplitude and area as an XRD peak. Note that many of the XRD peaks from the shock simulations required the superposition of multiple nearby pseudo-Voigt curves because of the complex stress gradients induced into the microstructures.

Table S4: Parameters for the pseudo-Voigt curve fits of Ta XRD plots: center (C), normalized amplitude (A), full-width-half-max width (FWHM), integral-breadth width (IB).

Time (ps)	C (°)	A (a.u.)	FWHM (°)	IB (°)
0	38.483	1.0	0.288	0.444
20	41.572	1.0	1.433	2.156
	42.156	0.421	1.163	1.745
50	38.323	1.0	0.972	1.445
	38.821	0.258	0.637	0.678
94	37.361	1.0	1.249	1.823
	37.967	0.236	1.253	1.547
	35.666	0.124	1.015	1.595

Table S5: Parameters for the pseudo-Voigt curve fits of Fe XRD plots: center (C), normalized amplitude (A), full-width-half-max width (FWHM), integral-breadth width (IB).

Time (ps)	C (°)	A (a.u.)	FWHM (°)	IB (°)
0	44.437	1.0	0.181	0.278
20	48.629	1.0	1.819	2.712
	46.103	0.569	1.264	1.866
	43.121	0.274	1.487	2.186
42	43.98	1.0	1.575	2.331
	44.358	0.667	0.752	0.958
71	42.487	1.0	1.781	2.635
	43.631	0.354	1.512	2.2
Fe HCP	48.621	1.0	0.248	0.388
	42.349	0.539	0.122	0.182
	46.636	0.369	0.227	0.243

Table S6: Parameters for the pseudo-Voigt curve fits of Cu XRD plots: center (C), normalized amplitude (A), full-width-half-max width (FWHM), integral-breadth width (IB).

Time (ps)	C (°)	A (a.u.)	FWHM (°)	IB (°)
0	43.102	1.0	0.26	0.398
	50.252	0.962	0.125	0.201
20	46.328	1.0	1.09	1.676
	53.509	0.214	2.34	3.509
50	42.778	1.0	1.18	1.921
	43.197	0.324	0.64	0.988
	49.191	0.244	2.785	4.205
75	41.759	1.0	1.25	1.857

	41.156	0.626	2.375	3.467
	47.363	0.355	3.845	5.414

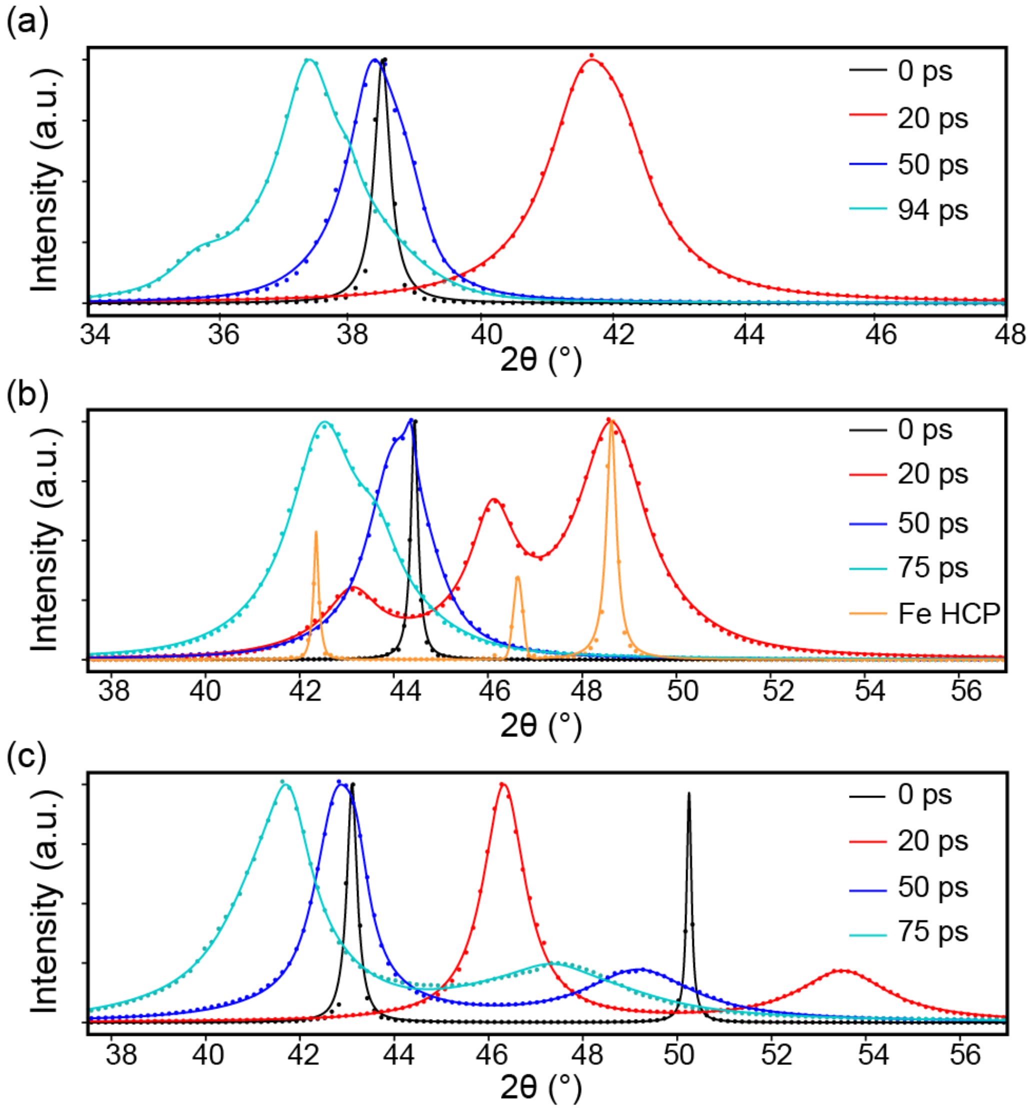

Figure S2: XRD for (a) shocked Ta, (b) shocked Fe, and (c) shocked Cu at time steps corresponding to the initial state, compression, release, and tension.

Note 3: Selected Microstructures

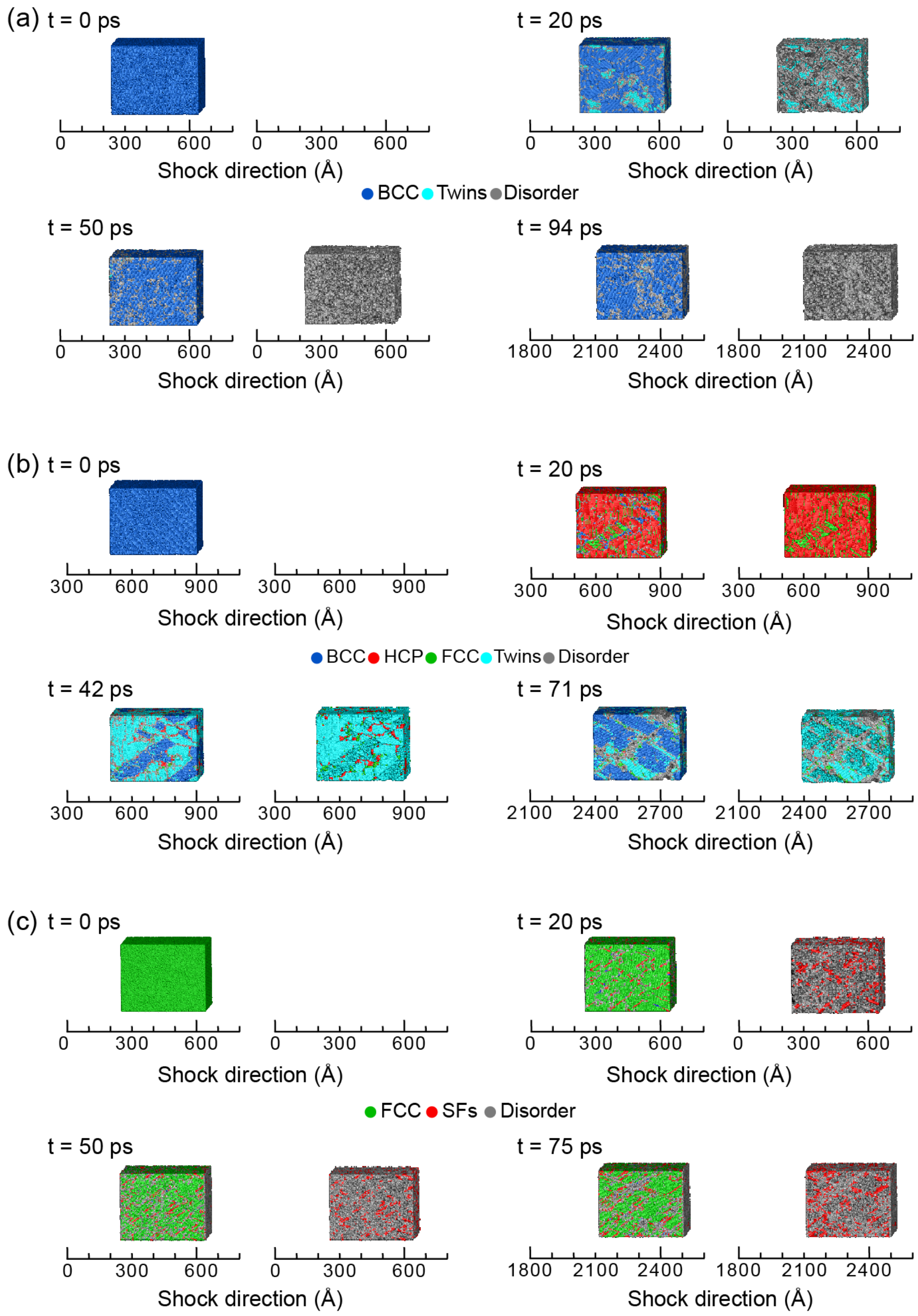

Figure S3: Cropped microstructure with and without the ground-state atoms (BCC atoms for Ta and Fe, and FCC atoms for Cu) for (a) shocked Ta, (b) shocked Fe, and (c) shocked Cu at time steps corresponding to the initial state, compression, release, and tension.

Supplementary References

1 Ida, T., Ando, M. & Toraya, H. Extended pseudo-Voigt function for approximating the Voigt profile. *Journal of Applied Crystallography* **33**, 1311-1316, doi:10.1107/S0021889800010219 (2000).

2 Kunka, C., Boyce, B. L., Foiles, S. M. & Dingreville, R. Revealing inconsistencies in X-ray width methods for nanomaterials. *Nanoscale* **11**, 22456-22466, doi:10.1039/c9nr08268a (2019).
